# Activity-based cost analysis of contrast-enhanced ultrasonography (CEUS) related to the diagnostic impact in focal liver lesion characterisation

**DOI:** 10.1007/s13244-015-0402-4

**Published:** 2015-05-09

**Authors:** Arianna Lorusso, Emilio Quaia, Gabriele Poillucci, Fulvio Stacul, Guido Grisi, Maria Assunta Cova

**Affiliations:** U.C.O. di Radiologia, Dipartimento di Scienze Mediche, Chirurgiche e della Salute, Università degli Studi di Trieste, Azienda Ospedaliero – Universitaria AOUTS, Ospedale di Cattinara, Strada di Fiume 447, 34149 Trieste, Italy

**Keywords:** CEUS, Focal liver lesions, Activity-based cost analysis, Diagnostic Impact, Economic evaluation

## Abstract

**Purpose:**

This study was done to assess the clinical-diagnostic impact and cost of contrast-enhanced ultrasound (CEUS) versus computed tomography (CT) and magnetic resonance (MR) imaging in the characterisation of focal liver lesions.

**Materials and methods:**

CEUS with sulphur hexafluoride-filled microbubbles (SonoVue bolus 2.4 ml) was performed in 157 patients with 160 focal liver lesions identified by other diagnostic techniques. CEUS images were obtained during the arterial (15 to 35 s from contrast injection), portal venous (40 to 70 s) and late phase (up to 300 s from microbubble injection). Contrast-enhanced CT was performed with a 64-row multidetector CT. MRI was performed before and after administration of the liver-specific contrast agent gadobenate dimeglumine (Gd-BOPTA). A patient-by-patient activity-based cost analysis was performed.

**Results:**

CEUS led to a change in the diagnostic workup in 131/157 patients (83.4 %) and in the therapeutic workup in 93/157 patients (59.2 %). CEUS allowed for the final diagnosis to be established in 133/157 patients (84.7 %). The full cost of CEUS was lower than that of contrast-enhanced CT and MR imaging.

**Conclusions:**

CEUS determined a change in the diagnostic and therapeutic workup in the characterisation of focal liver lesions and reduced the full costs of the diagnostic process.

***Main messages*:**

*• CEUS allows a correct diagnosis in more than 80* % *of focal liver lesions*.

*• CEUS has a significant impact on the diagnosis of focal liver lesions*.

*• CEUS examination of focal liver lesions reduces total costs*.

*• Dynamic MR with hepato*-*specific contrast medium remains the reference standard for lesion characterisation*.

*• CEUS is low*-*cost*, *versatile and accurate in the characterisation of focal liver lesions*.

## Introduction

Greyscale ultrasound (US) in the study of the liver is a noninvasive, inexpensive and readily accessible technique. In spite of continuous technological advancements, ultrasound is characterised by low specificity in the study of focal hepatic lesions and has an overall accuracy of about 50 % [1]. Colour Doppler and pulsed Doppler modules provide useful supplementary information for characterising focal hepatic lesions, with limitations related to breathing and cardiac pulsatility artefacts, allowing only the study of the macrocirculation, as smaller vessels cannot be visualised [2, 3].

Contrast-enhanced US (CEUS) is now recognised as a highly accurate test in the detection and characterisation of focal hepatic lesions [2, 4–6]. It is a minimally invasive (considering the required venous access), repeatable technique that is readily available in the ultrasound suite, has high contrast and temporal resolution, and allows dynamic evaluation of lesions in real time [2–7].

CEUS has comparable sensitivity and specificity to computed tomography (CT) and magnetic resonance imaging (MRI), albeit with limitations related to the lack of panoramicity and the physical impediments to ultrasound penetration in the presence of obesity and bowel gas [9–11].

Only a limited number of studies in the literature have carried out a cost analysis of hepatic CEUS [12–15].

The aims of our study were to evaluate the clinical-diagnostic impact of CEUS for the characterisation of focal hepatic lesions in healthy, cirrhotic and oncologic patients and to analyse the cost of CEUS in comparison to multiphase CT and dynamic MR imaging with a hepato-specific contrast agent.

## Materials and Methods

### Patients

A prospective study was conducted on a consecutive series of 157 patients (74 males and 83 females; age range, 30–92 years; mean age, 61.4 years) with 160 focal liver lesions examined between 30 March 2010 and 30 March 2011. All subjects gave their informed consent to undergo the imaging procedure.

There were 80 patients with a normal liver, 44 patients with a known primary malignancy and 33 patients with liver cirrhosis. In our radiology department CEUS is used as a second-level technique after detection of focal liver lesions in both the normal and cirrhotic liver according to the 2004 and 2011 EFSUMB guidelines [16, 17]. In fact, the study included all patients who underwent liver CEUS as a second-level investigation for the characterisation of focal lesions detected on other imaging techniques and considered indeterminate by the operator; in more detail, in 74 patients CEUS was carried out after baseline ultrasound, in 77 after CT, in 5 after MR imaging and in 1 after scintigraphy (Indio-111 octreotide).

As for the lesions detected on unenhanced US, these were newly discovered focal lesions in patients with a known primary malignancy in 9 cases, suspicious findings for malignancy in patients with cirrhosis in 22 cases, newly detected findings that could not be characterised on unenhanced US in 11 cases, focal liver lesions discovered incidentally during unenhanced US examinations performed in an emergency setting in 12 cases and focal lesions identified but not characterised at another institution in 20 cases.

As for the indeterminate focal liver lesions detected with CT, 32 were identified during a staging or follow-up examination performed with a single-contrast phase in patients with known malignancy, 10 were suspicious lesions in patients with cirrhotic livers, 7 were incidental findings on CT angiography (*n* = 4) or CT urography (*n* = 3), 17 were incidental findings during single- or dual-phase emergency CT, 5 were lesions presenting an atypical dynamic contrast pattern on multiphase CT, and 6 were lesions detected with single- or dual-phase CT at other institutions/hospitals and not characterised.

Of the five cases of CEUS performed after MR imaging, 2 had MR findings compatible with atypical haemangioma in high-risk patients, two required differentiation between well-differentiated hepatocellular carcinoma (HCC) nodules and benign lesions, and one was a request for further contrast-enhanced investigation to confirm a suspicion of single metastasis in a cancer patient (known malignancy).

The single patient who underwent CEUS after positive scintigraphic examination (patient with colon carcinoma and focal uptake in the liver area) underwent CEUS to remove any doubts relating to the finding.

### Examination technique

Liver CEUS was conducted with Sequoia S2000 equipment (Acuson-Siemens, Mountan View, CA) in 133 patients and with an Aplio XG system (Toshiba Medical Systems, Tokyo, Japan) in 24 patients. After performing an initial baseline greyscale study and colour and/or power Doppler imaging with multifrequency convex probes in order to identify the lesion and select the best scanning plane, we proceeded to cannulate a forearm vein and carry out the CEUS study. A bolus of sulphur hexafluoride microbubbles (SonoVue ® Bracco Imaging, Milan, Italy) was injected at an average dose of 2.29 ml (mode and median, 2.4 ml) followed by a 10-ml saline bolus chase. The arterial phase was set at 15–35 s from contrast administration, the venous phase at 40–70 s and the late phase up to 300 s. The contrast-specific techniques used were cadence contrast pulse sequencing (CPS) (Acuson-Siemens, Mountan View, CA) and a tissue contrast discriminator (Toshiba Medical Systems, Tokyo, Japan) with a mechanical index of 0.09–0.14, a dynamic range of 65 dB and a temporal resolution between 75 and 100 ms (10–13 frames per second). Signal amplification was adjusted below visibility of noise and the focus immediately below the lesion.

Four-phase CT was conducted using 64-slice equipment (Aquilion, Toshiba Medical Systems, Tokyo) with the following technical parameters: 400-ms rotation time, 64 × 0.5-mm collimation, pitch normalised at 1, 32-mm Z-axis coverage, 0.3-mm reconstruction interval, 120 kV, 180–250-mAs tube current intensity in relation to patient size and 40-cm field of view. The images were reconstructed with a field of view of 25–35 cm in relation to the physical constitution of the patient. We acquired unenhanced, arterial, portal and late phase scans after the intravenous bolus administration of 120 ml of iodinated contrast medium (iopromide) at a concentration of 370 mg/ml at a flow rate of 5 ml/s, followed by a 50-ml saline bolus. The arterial phase was acquired using the bolus tracking technique with a delay of 18 s after a threshold of 140 HU had been reached in a region of interest (ROI) placed over the abdominal aorta. The portal and late phases were acquired with a delay of 70–80 s and 180–210 s, respectively, from the beginning of contrast administration.

MR of the liver was performed using a superconducting magnet operating at 1.5 T (Achieva, Philips Medical Systems, The Netherlands). Axial acquisitions were obtained during an expiratory breath-hold using a four-channel phased-array surface coil and breathing synchronisation. The following sequences were used: T2-weighted single-shot turbo spin-echo (SS TSE) (TR/TE, 593/80), T2-weighted inversion recovery with fat suppression (SPAIR) (TR/TE, 448/80), in-phase and out-of-phase T1-weighted fast field echo (FFE) (TR/TE, 332/4.6-2.3) and T1-weighted high-resolution isotropic volume examination (THRIVE) with fat suppression (TR/TE, 3.2/1.62) performed before and after the administration of 0.2 mmol/kg gadobenate dimeglumine (Gd-BOPTA) into a vein of the arm at a flow rate of 2 ml/s and followed by 20 ml of saline bolus chase. The arterial phase was acquired using the bolus chase technique with a delay of 5–10 s after visualisation of the contrast medium at the level of the abdominal aorta. The portal and late phases were acquired with a delay of 70–80 and 180–210 s, respectively. Finally, a hepatobiliary phase was acquired at 1.5–2 h after the administration of contrast medium.

### Image evaluation

The images were evaluated on a PACS (picture archiving and communications system)-integrated workstation (19-inch TFT display, resolution 2,560 × 1,600 pixels, EBIT AET Health, Genoa, Italy) by the radiologist who performed the examination. In particular, three radiologists with 5, 14 and 15 years of experience in liver imaging reviewed the CEUS images, another three radiologists with 9, 13 and 14 years of experience reviewed the CT images, and two radiologists with 9 and 12 years of experience reviewed the MR images.

Evaluation of the CEUS images was performed in real time, whereas the CT images were reviewed just after the acquisition and reconstruction of the four phases (unenhanced, arterial, portal and late phase); the characterisation of focal liver lesions on MR imaging required waiting until after the acquisition of the hepato-specific phase 60 min after administration of the contrast medium.

We used the reference criteria reported in the literature for CEUS [18] and MR imaging and CT [3] for lesion characterisation or to propose a possible nature diagnosis, benign or malignant, for each focal liver lesion.

### Cost analysis

For the purposes of the economic assessment we used a method already applied in previous studies [19] and in industrial settings [20] to compare the relative costs of the three imaging techniques used for the characterisation of liver lesions: CEUS, CT and MR imaging.

For CEUS, data were collected for 157 patients. Given that only a small percentage of these patients required subsequent assessment with multiphase CT (*n* = 6 ) and/or dynamic MR imaging with hepato-specific contrast material (*n* = 13), the variable costs and personnel costs for these two modalities were calculated on a sample of 50 patients with focal liver lesions.

For each technique, we calculated and compared the full cost, defined as the sum of the variable costs and fixed costs of capacity (technology and staff costs), which represents the most relevant cost component as it differs among CEUS, CT and MR imaging; the indirect costs of the department were not taken into account as they do not depend on the application of the three techniques.

For each method, the cost of the technology was calculated based on the purchase cost of the equipment, obtained from the official hospital documentation, and depreciation over time considered at a constant annual rate except in cases in which the degradation of the device was available. The useful economic lifespan of the equipment was defined as 10 years, i.e., the maximum time that includes both technological obsolescence and degradation of care efficacy.

For each investigation, we then assessed the variable costs relating to the type and quantity of materials and services related to its use.

Subsequently, we evaluated the personnel costs in terms of physician time (person responsible for the activity, including staff radiologists and trainee radiologists) and radiology technician and nursing staff time, considering the specific activities undertaken by the various professionals for each of the three imaging techniques; this provided a calculation of both fixed and variable costs closely reflecting the current situation at our university hospital.

The costs of fixed asset utilisation (technology and staff costs) were obtained from time measurements of the use of capacity that these production factors offer.

The calculated costs of materials and equipment are inclusive of value added tax at 20 %.

Then we calculated the common costs, which correspond to the internal costs of the production factors of the radiology division and are necessary for providing services common to all diagnostic activities carried out within the division. These include capacity costs related to personnel and support materials, which remain constant, regardless of the total number of examinations performed in a year.

Finally, we considered the external costs to the radiology division for each of the imaging techniques being compared: in particular, the costs arising from determination of serum creatinine levels (performed in all patients undergoing CT or contrast-enhanced MR imaging) and those arising from anti-allergic preparations through the use of corticosteroids associated with anti-H1 and anti-H2 (performed in all patients undergoing CT with a history of moderate and severe reactions to iodinated contrast media or a history of asthma or allergy requiring medical treatment).

The total cost for each investigation, and hence the cost of the diagnostic process, is given by the sum of the full costs, common and external costs.

We computed the actual historical full cost for the three different imaging techniques, CEUS, CT and MR imaging, which corresponds to average costs. We followed an activity-based method [36] consisting of two tasks: producing the images and reporting. In our case the second task closely follows the first one; it is performed in the same cost and responsibility department, and its cost is computed separately (as lead time times cost per hour); therefore, the two tasks are additive.

The economic evaluation method is cost-effectiveness [20], namely the diagnostic effectiveness, as an intermediate time of the whole treatment process. We assumed the provider point of view for the costs. We excluded the costs for the patient, the patient’s family or discounted future effects for the patient.

## Results

### Clinical-diagnostic impact

**Fig. 1:**
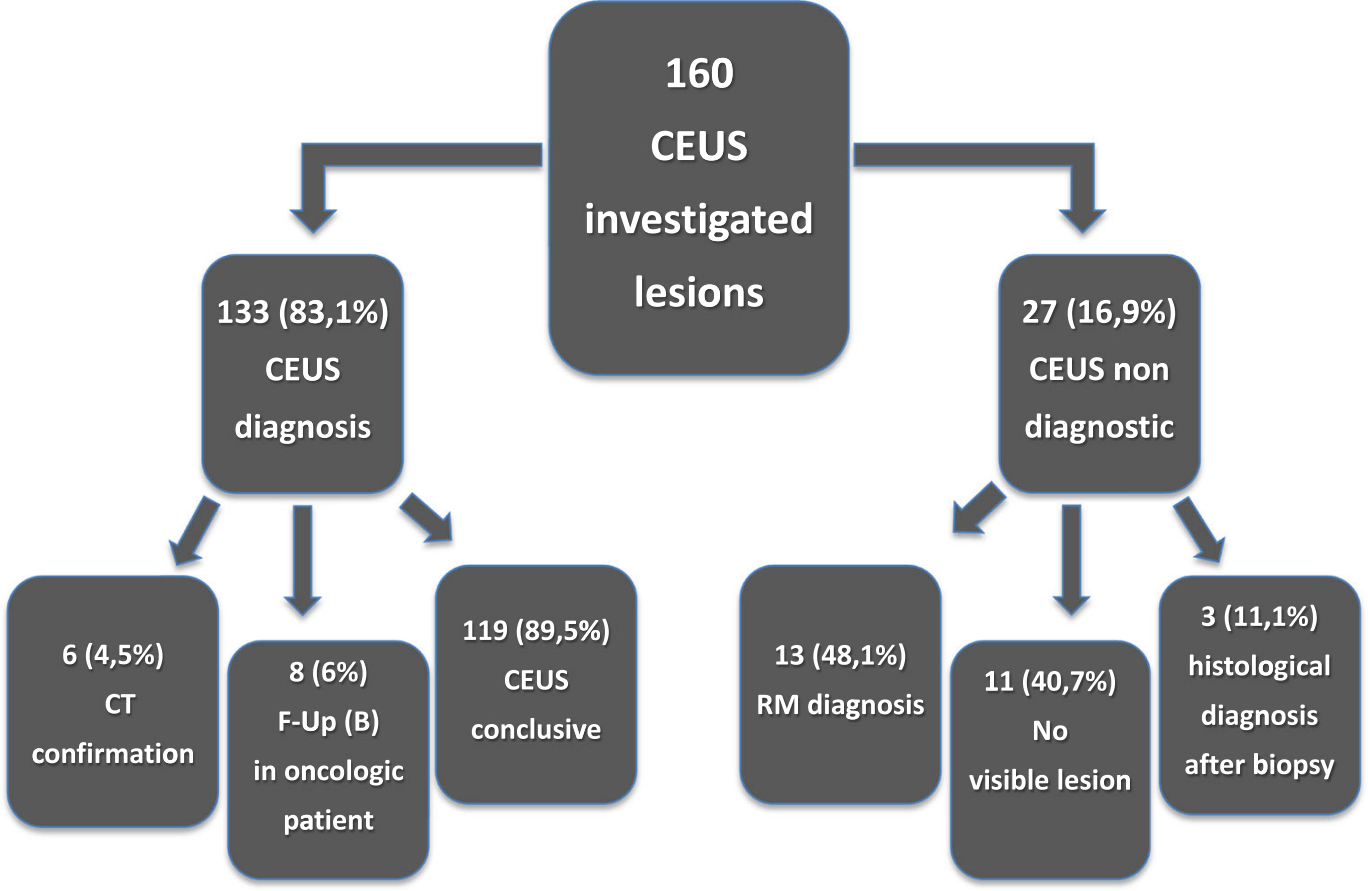
Percentage of lesions suspicious or characterised as benign or malignant after CEUS examination

**Fig. 2:**
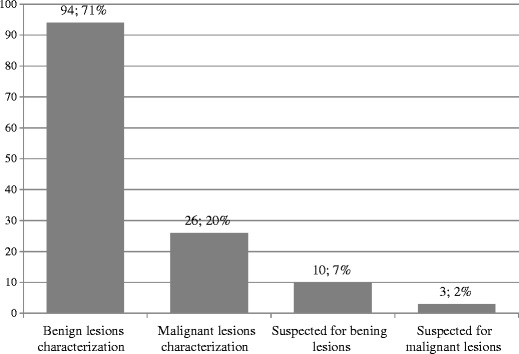
Total number and percentage of lesions suspected or characterised as benign or malignant though CEUS

Table 1 shows the overall diagnostic impact provided by CEUS in our series. CEUS helped to propose a correct diagnosis in 133 out of 160 focal liver lesions investigated (see Fig. 1 for further details), providing a diagnosis of benign lesions in 94/133 cases (70.7 %) and malignant lesions in 26/133 cases (19.5 %) as well as a correct suggestion toward a benign or malignant lesion nature in 10/133 (7.5 %) and 3/133 cases (2.3 %), respectively (Fig. 2). The 120 liver lesions that were correctly characterised by CEUS were: 46 angiomatous lesions, 20 areas of macrovesicular steatosis, 16 cystic lesions, 7 regenerative nodules, 3 focal areas of altered perfusion, 1 case of focal nodular hyperplasia (FNH), 1 case of wound abscess following cholecystectomy, 14 HCCs and 12 metastases.

**Table 1:** The overall clinical impact provided by contrast-enhanced US in 157 focal liver lesions

Patients examined with CEUS	Lesions identified through imaging modalities other than US	Change in the diagnostic workup	Change in the therapeutic strategy
157	157/157	131/157	93/157
	100 %	83.4 %	59.2 %

### Economic evaluation

Table 2 shows the results of economic analysis of CEUS, CT and MR imaging.

**Table 2:** Cost analysis of the three imaging techniques: CEUS, multiphasic CT and dynamic MRI

	CEUS	CT	MR imaging
Full costs (€)			
Equipment	1.88	20.74	36.40
Variable costs	40.26	79.82	67.77
Medical doctors	31.20	24.89	37.86
Residents	6.80	1.96	4.56
Radiographers	0	10.39	20.79
Nurses	0	9.63	0
Common costs (€)	5.3	5.3	5.3
External costs (€)	0	2.3	1.9
Total costs (€)	€85.44	€ 155.03	€ 174.58

### Contrast-enhanced ultrasound

1. Equipment costs (depreciation and maintenance)
  The analysis considered the Sequoia Acuson S2000 ultrasound system (Siemens Mountan View, CA), for which the cost to be amortised (i.e., purchase cost minus residual value, here set at 0) was €117,120.00. The annual cost over 10 years’ amortisation was €11,712 calculated according to the equipment life span. Considering 300 days of practical annual capacity, a single daily 6-h shift in the ultrasound section and an average CEUS examination time of 17.4 min (measured on 157 patients imaged with CEUS), the equipment costs amounted to €1.88 per examination.
2. Variable costs (materials and related services)
  The performance of liver CEUS involves the use of sonographic contrast material (SonoVue, Bracco) in 4.7-ml bottles (€80.99). The average dose administered per patient was equal to 2.3 ml, for a cost of €39.63. Additional materials were a 10-ml vial of saline solution (€0.065), 20-G Abbocath needle cannula (€0.32 ), 10-ml sterile disposable syringe (€0.066 ), injection stopper (€0.041), ultrasound gel (cost per jar €0.51, at an average of 15 examinations per jar, €0.034 per examination) and disposable gloves (one pair for the radiologist performing the survey and another for the trainee radiologist, for a total cost of €0.067). In addition, outpatients, who account for 6–7 % of the liver CEUS examinations performed at our institution, receive a CD-ROM with the images (€0.32).
  The sum of the costs resulted in a variable cost of €40.18 per examination.
3. Staff costs
  The actual time of activity for a radiologist is equal to 175.95 days/year with an effective daily shift of 7.6 h. This results in an hourly cost of €75.80 and a cost per minute of €1.26.
  On the basis of 157 examinations performed, we measured the average procedure time to be 17.3 min and the average reporting time to be 7.4 min, for a total average radiologist time of 24.7 min per patient. Multiplying the cost/minute of the radiologist (€1.26) by the average time per patient (24.7 min) gives a “radiologist cost” of €31.20 per examination.
  As for the cost of trainee radiologists (residents), who are required to participate actively in CEUS investigations at our hospital, we considered an average of 210 working days per year, estimated based on specific provisions of national, European and international research programmes, and calculated subtracting ordinary leave, extraordinary leave and sick leave (5 %). We therefore considered an average of 7.2 h per day for 210 working days per year at an hourly cost of €16.70 and €0.28 per minute. The resident physician has a role in patient preparation, illustration of informed consent, vein cannulation and administration of the contrast medium. The average trainee radiologist time is equal to the average procedure time (17.3 min), plus the time required to explain the examination and take the vein (7 min), for a total of 24.3 min. The result is a “trainee radiologist” cost of €6.80 per examination. Thus, the full cost of a CEUS examination is €80.14.

### Multiphase CT

1. Equipment costs (depreciation and maintenance)
  The analysis considered a multislice CT Aquilion 64 scanner (Toshiba Medical Systems, Tokyo). The cost to be amortised is equal to the purchase cost (without subtracting a residual value) and is €1,242,000. This results in an annual cost of €124,200 for a 10-year amortisation. To this we must add the cost of the maintenance contract, amounting to €99,360 per year, and the cost of the injection pump (including routine annual maintenance) of €4,056 (equal to €405.6 per year). The sum of the annual depreciation over 10 years and the annual cost of the maintenance contracts results in an annual equipment cost of €223,966.
  Assuming 300 days of practical capacity, the daily cost is €746.6. Assuming an hourly cost of €62.21 with a single daily 12-h shift in the CT section and an average CT investigation time of 20 min, the equipment costs amount to €20.74 per examination.
2. Variable costs (materials and related services)
  Performance of a hepatic multiphase CT involves the use of 120 ml of iodinated contrast medium at a concentration of 370 mgI/ml (iopromide) in 500-ml bottles (€159.76), for a cost of €38.34.
  Additional materials include 30-ml bottles of saline solution (negligible cost of €0.31 per 250 ml), a set of disposable syringes for the injection pump with a unit cost of €24, 20-G Abbocath (€0.32), spiral connector (€2.95) and pair of disposable gloves (€0.037). Costs for the disinfectant, cotton ball and plaster are negligible. Outpatients (about 28 %) are also given a CD-ROM of the images (€0.32). The result is a cost per examination of €0.084.
  Also included in the analysis was the wear of the X-ray tube (this is a depreciation calculated according to use and thus a variable cost): we calculated that the average number of shots per scan is 45. The purchase cost of the X-ray tube is €91,800 and its expected life is equal to 300,000 shots, with a cost of €0.30 per shot, for a cost of €13.77 per examination.
  The sum of the values results in a variable cost of €79.82 per examination.
3. Staff costs
  Radiologist time is related to the various activities to be carried out: evaluation and planning of the investigation in relation to the clinical question (principle of justification), monitoring and evaluation of images in the different contrast-enhanced scans, comparison with previous examinations and reporting.
  On this basis, the analysis carried out on a sample of 50 patients showed that multiphase CT of the liver involves a mean radiologist time of a total of 19.7 min, equal to a “radiologist cost” of €23.52 per examination.
  At our hospital, trainee radiologists are also required to attend CT examinations, where their role is to correctly explain the procedure, collect the patients’ history of allergy to iodine-based compounds, verify laboratory tests (creatinine) and provide the patient with all the information needed for informed consent to the procedure. The average trainee radiologist time is 7 min, with a resulting “trainee radiologist cost” amounting to €1.96.
  Every multiphase CT study involves the use of 20-min technician time.
  The annual cost of the radiology technician amounts to €40,078.52. Subtracting from the annual working hours absences due to ordinary leave (44 days), extraordinary leave and sick leave (average 30 %), a technician’s actual working time is 178.5 days of 7.2 productive hours. The cost per hour being €31.18 and the cost per minute €0.52, we have a “radiology technician cost” equal to €10.39 per examination.
  The CT examination must also be attended by a nurse who has a role in patient preparation and contrast material administration. In our sample, the average time needed for nurses to perform these activities (i.e., patient reception and preparation, vein cannulation) was equal to 20 min.
  We considered an annual cost of €39,256 per nurse. Annual ordinary leave for nurses is 32 days a year. Nurses work 35 weeks per year and 36 h per week. Subtracting the annual ordinary and extraordinary leave, we have a total of 188.7 working days of 7.2 productive hours. This yields a cost per hour equal to €28.89 and a cost per minute of €0.482. The “nursing staff cost” is therefore €9.63 per examination.
  The full cost of a multiphase CT examination of the liver at our institution is therefore €149.73.

### CE dynamic MR imaging

1. Equipment costs (depreciation and maintenance)
  We considered the current cost of an Achieva 1.5-T MR device (Philips Medical System, The Netherlands). The cost to be amortised (equal to the purchase cost) is €1,000,000. This results in an annual cost of €100,000 for a 10-year amortisation. To this we added the costs of maintenance, amounting to €94,758 per annum. The purchase cost of the injection pump is €18,000 with an annual cost of €1,800. The routine maintenance of the pump at our institution is the responsibility of Clinical Engineering, so the cost is not incurred by the Division of Radiology.
  The sum of the annual depreciation for 10 years and annual costs for maintenance contracts results in an annual equipment cost of €196,558. Assuming 300 days of practical capacity, the daily cost is €655.19. Considering an hourly cost of €54.6 with a single 12-h daily shift, and an equipment time of 40 min for this investigation (30 min for the first part of the acquisition and another 10 min for the late acquisition starting 1 h after contrast administration), the result is a “total equipment cost” of €36.4.
2. Variable costs (materials and services)
  At our institute, dynamic MR imaging of the liver involves the use of the hepato-specific contrast medium Gd-BOPTA (gadobenate dimeglumine) in 20-ml bottles (€57.07). In our sample, an average of 14 ml was used, for a cost of €39.95. Further materials include: approximately 20 ml of saline solution whose cost is negligible; a set of disposable syringes for the injection pump with a unit cost of €24; a spiral connector with a cost of €2.95; a 20-G Abbocath (€0.32); two pairs of disposable gloves (€0.074); a small amount of disinfectant; a cotton wool ball and a bandage. Outpatient referrals, which account for approximately 33.3 % of requests for liver MRI studies performed at out institution, are provided with a CD-ROM of the images.
  The variable cost is therefore €67.77 per examination.
3. Staff costs
  Radiologist working time is related to the various activities performed in the MR section: investigation approval and settings, evaluation of any previous imaging investigations and evaluation of scans acquired during the different post-contrast phases, and reporting. In addition, in dynamic liver MR imaging, also the hepato-specific phase (the late acquisition at 1 h after administration of contrast material) is assessed.
  In the sample of 50 patients who underwent the survey, we estimated an average radiologist time of 29.97 min, for a cost of €37.86.
  At our hospital, the MR unit is always attended by a trainee radiologist who has a role in explaining the modality, assessing patient compatibility by administering a questionnaire and obtaining informed consent. Trainee radiologists are also involved in vein cannulation.
  Based on an average trainee radiologist time of 16.3 min, the “trainee radiologist cost” is €4.56.
  Each liver MR imaging investigation involves about 30 min technician time for the first stage and an additional 10 min for the hepato-specific phase 1 h after administration of the contrast agent. This results in a total technician time of 40 min, which, multiplied by the cost/minute, provides a “radiology technician cost” of €20.79.
  The full cost of a dynamic liver MR imaging investigation at our institution is therefore €169.34.

#### Common costs

“Common costs” are taken to refer to the cost of production factors internal to the radiology division and involved in generating a wide range of services common to all diagnostic activity performed within the division. These are capacity costs that do not vary despite even major changes in the total number of examinations carried out over the year. Common costs are costs pertaining to the support staff, i.e., all those professionals not mentioned above who work to help run the institute regardless of the type of examination (head of division, chief radiology technician, auxiliary staff, archive technicians, administrative clerks and assistants) and support materials (computers, printers, recorders, electrical and network equipment, stationery, cleaning supplies). Over the year, the total cost for these workers was €601,735 and the total number of examinations was 126,592, resulting in an additional common cost of €4.8 per examination. The cost of the support material was estimated to be €0.50 per procedure. Therefore the total cost of personnel and support material amounted to €5.30.

#### External costs

All patients undergoing a CT investigation involving the use of iodine-based contrast material routinely undergo blood testing to assess serum creatinine levels. This also applies to those undergoing MR imaging with the administration of the paramagnetic contrast agent. This implies an external cost of €1.90 per examination for both multiphase CT and contrast-enhanced dynamic MR imaging.

In addition, 1.1 % of patients undergoing CT have a history of allergy that requires pharmacological prophylaxis (based on corticosteroids associated with anti-H1 and anti-H2) at a cost of €4, leading to an additional €0.40 on average for each examination.

Overall, these external costs amount to €0 for CEUS, €2.3 for multiphase CT and €1.9 for dynamic MR imaging.

#### Cost of the diagnostic process

The sum of the full costs, common costs and external costs provides the cost of the diagnostic process for each imaging modality taken into consideration (Table 2).

Considering that in our current economic context the item that most affects the full cost of a modality is the cost of technology, we compared the full cost of the three imaging techniques, assuming the most unfavourable condition for CEUS, that is, an up to 40 % decrease in the cost of technology (equipment cost) of CT and MR imaging with unchanged cost of US equipment.

## Discussion

As is well established in the literature [3–9], CEUS is an easy-to-perform investigation that has high diagnostic accuracy in the characterisation of focal liver lesions. According to the EFSUMB guidelines [16, 17] CEUS should be used in each incidental focal liver lesion detected at unenhanced US, in lesions or suspected lesions detected with unenhanced US in patients with a known history of malignancy, when CT or MR imaging is inconclusive, and to characterise all nodules found on surveillance with US.

This is also evident from our results, as CEUS liver imaging, performed as a second-level examination, thanks to the reference diagnostic patterns reported in the literature [16, 1, 2, 4, 21, 22], proved to be diagnostic in 83.1 % of the lesions investigated and allowed for lesion characterisation (without the need for further imaging confirmation or follow-up) in almost 90 % of these cases.

The CEUS survey is also much more objective than baseline US: the operator must in fact observe the enhancement behaviour of the lesions during the different post-contrast phases to achieve correct characterisation without having to take into account the complex semeiotics of conventional US [1, 2, 4–7]. In addition, the correct performance and interpretation of a CEUS examination requires experience as well as adequate theoretical and practical training. CEUS is considered a completion of unenhanced US, the semiotics of which the operator should know equally well. CEUS is an investigation that requires appropriate equipment and the use of contrast-specific techniques [2, 23].

The data from our series, collected at a specific moment in time in the setting of our department of radiology, reveal two important aspects: in more than 80 % of the 157 patients investigated, CEUS led to a real change in the diagnostic workup that the clinician, after discussion with the radiologist, would have followed if CEUS had not been available; in almost 60 % of cases, CEUS was able to determine a real change in the clinical and therapeutic management of the patient.

These data suggest that the technique has a major impact on the clinical management of patients. It must be emphasised that this conclusion is based on the clinician’s evaluation, taking into account the impact of CEUS on daily activity, but in the absence of a “gold standard”.

CEUS therefore appears to be a “problem-solving” technique able to orientate the diagnosis towards the benignity or malignancy of a lesion that could not be characterised on other imaging techniques with a high level of diagnostic accuracy [4, 24–27].

In our series, only a limited number of patients could be imaged with CEUS immediately after detection of the equivocal finding, as this depended on the availability of an adequately experienced operator; in the majority of patients CEUS was scheduled for a later date.

CEUS has the potential immediate effect of shortening the diagnostic workup of patients with a newly detected focal liver lesion by avoiding the need for more expensive additional investigations, which are usually associated with longer waiting times.

These aspects have been considered by several studies in the literature: Faccioli et al. [12] conducted a study on 398 patients showing that CEUS is the most economical second-level technique for the diagnosis of benign focal liver lesions, allowing cost savings in the management of internal resources; Romanini et al. [13] evaluated a series of 485 patients with 575 focal liver lesions and showed that the routine use of CEUS for focal liver lesion characterisation leads to significant cost savings while providing a diagnostic accuracy comparable to CT and MR imaging, and allows the diagnostic process to be concluded in 87.6 % of cases without the need for additional imaging.

More recent studies have reported similar findings. In particular, Tranquart et al. [14] reported a clear economic advantage for CEUS compared to CT and MR imaging, confirming the high diagnostic value of CEUS in the characterisation of focal liver lesions. A similar conclusion was reached by Sirli et al. [15].

In settings in which the technique and expertise are available, CEUS can be performed in real time immediately after detection of the finding. Given that the majority of incidental focal liver lesions are benign, this could avoid unnecessary extensions of the diagnostic workup, further reducing waiting times or length of hospital stay and, consequently, costs.

Our study also found that CEUS often provides an additional diagnostic value for those focal liver lesions that cannot be characterised on CT. This may happen in incidental focal liver lesions presenting atypical enhancement or identified on single-phase CT (usually portal phase), or when the amount of iodinated contrast medium injected per minute is not appropriate to the patient’s body weight resulting in low contrast in the liver, in small lesions in which evaluation of enhancement proves difficult and in patients with inhomogeneous liver echogenicity or also after chemo- or radiotherapy.

In these cases, there is agreement in the literature [2, 8, 10, 18–22, 26–29] that CEUS represents an additional diagnostic value thanks to its greater temporal resolution and its limited field of view concentrated in the evaluation of a particular sector of the liver with an resolution comparable to CT and MR imaging.

The cost analysis performed in our study aimed to compare the three imaging methods used for the characterisation of focal liver lesions. Economic analysis is used to identify and make explicit a set of criteria that can help in the process of choosing between different resources. The main task of any economic evaluation is to identify, measure, estimate and compare the costs and consequences of the various alternatives considered [20]. New technologies, in particular at the time of their introduction into clinical practice, may have high costs that can be justified by demonstrating a real diagnostic and therapeutic impact. The effects of technological innovation on health spending depend solely on its appropriate use, considering the diagnostic and/or therapeutic strategies already available. These considerations are even more valid in radiology as the costs of the devices and their use have a significant impact on the total costs. In addition, as also noted in our series, the radiological diagnosis significantly affects the patient’s clinical management, treatment choices and consequently the overall cost of the disease [30–33].

Our cost analysis of the three techniques used for liver imaging—namely, CEUS, multiphase CT and contrast-enhanced dynamic MRI—clearly shows that liver CEUS is the most advantageous in economic terms. This finding is no doubt related to the lower cost of the equipment, but also to the smaller number of professionals involved and the lower impact of variable costs (Table 2).

In addition, as shown in the sensitivity graph, even assuming the worst case scenario, that is, that the cost of the ultrasound scanner remains constant with maximum conceivable depreciation of the costs of CT and MR imaging technology, there is still a clear economic advantage for CEUS examination of the liver.

Our cost analysis was carried out using a method commonly applied in industrial settings, which guarantees a rigour that reduces approximations of conventional cost calculation methods [30]. This analysis provides, however, a snapshot of the situation of the Radiology Division at a particular moment in time, so the data cannot be extrapolated uncritically, as in different settings the costs may differ greatly as a result of both the equipment and materials available and organisational decisions affecting the cost of personnel.

It should, however, be remembered that CEUS does not have absolute diagnostic accuracy in the characterisation of focal liver lesions and that dynamic MR imaging with hepato-specific contrast medium remains the reference standard for lesion characterisation [3, 10, 22, 27, 29]. However, considering its high cost, the use of MR imaging must necessarily be restricted to a limited number of patients, i.e., those with focal liver lesions showing equivocal enhancement at CEUS, those with lesions in locations that cannot be explored with ultrasound or patients who are difficult to assess with ultrasound. However, microbubble contrast agent with an additional postvascular (or Kupffer) phase, such as Sonazoid, which is approved for liver imaging in Japan, could further increase the overall diagnostic accuracy for CEUS [34], especially in the cirrhotic liver [35].

Our study has some limitations. First, in our study we performed a detailed comparative assessment of the full (technology, variable and staff costs), common and external costs of the three modalities and evaluated the diagnostic and therapeutic impact of CEUS. However, our evaluation of cost-effectiveness cannot be considered complete in that we failed to assess the impact of the different diagnostic strategies on the patients’ survival and quality of life [28–31]. Second, the results of our cost analysis apply to our specific health care setting since there is a signicant variation across European countries in terms of salaries of medical doctors, nurses and radiographers, and also in costs of imaging equipment, contrast agents and SonoVue. Moreover, the low cost of CEUS, related to the examination time, applies to a health care setting where CEUS is well established since CEUS is not part of radiology training in many European countries.

In conclusion, liver CEUS represents a low-cost, versatile and accurate technique in the characterisation of focal liver lesions [5, 10, 13]. CT and MR imaging, while remaining reference techniques for the characterisation of focal lesions, are associated with longer waiting times and hospitalisation before providing a definitive diagnosis, with resulting additional costs. Our study confirms that CEUS provides a viable alternative to CT and MR imaging in the study of focal liver lesions and that it can reduce the costs associated with the use of more sophisticated diagnostic techniques by shortening the diagnostic process and patient hospitalisation.

## Conclusions

CEUS is an accurate diagnostic investigation in the characterisation of focal liver lesions and it reduces the costs associated with the use of more sophisticated diagnostic techniques.

## Abbreviations

CEUS: contrast-enhanced ultrasound
CT: computed tomography
